# Prevalence of thinness and its effect on height velocity in schoolchildren

**DOI:** 10.1186/s13104-021-05500-3

**Published:** 2021-03-16

**Authors:** Hassib Narchi, Afaf Alblooshi, Maisoon Altunaiji, Nawal Alali, Latifa Alshehhi, Huda Alshehhi, Asma Almazrouei, Ahmed R. Alsuwaidi, Abdul-Kader Souid

**Affiliations:** 1 Department of Paediatrics, College of Medicine and Health Sciences, United Arab Emirates (UAE) University, P.O. Box 17666, Al Ain, United Arab Emirates; 2 Department of Medical Education, College of Medicine and Health Sciences, United Arab Emirates (UAE) University, Al Ain, United Arab Emirates; 3 School Health Team, Ministry of Health, Ras Al-Khaimah, United Arab Emirates

**Keywords:** Underweight, Growth, Height, Obesity, Schoolchildren, Body mass index, Epidemiology, Prevalence

## Abstract

**Objective:**

In contrast to childhood obesity, studies involving thin children are much fewer, especially in developed countries. Furthermore, most reports do not address the impact of childhood thinness on height velocity. This study investigated the prevalence of thinness and its effect on height velocity in schoolchildren in the United Arab Emirates (UAE). Weight and height were measured in 29,410 schoolchildren (50.5% females), as part of the health assessment (academic year 2014–2015). The body mass index (BMI) was classified as normal, thinness, overweight, or obese using cutoffs established by the International Obesity Task Force (IOTF), World Health Organization, and Centers for Disease Control.

**Results:**

The median age was 10.2 years (range, 3–19). Using the IOTF scale, one-quarter of the children aged 4–6 years and one-third of the children aged 7–9 years were thin (BMI ≤ 18.5 kg/m^2^). Thinness was less prevalent (8–10%) in adolescents. Group peak height velocity was delayed 1–3 years in thin children and was higher in children with excess body fat. In conclusion thinness was the highest (25–33%) in children aged 4–9 years of age and their peak height velocity was delayed 1–3 years when compared to the other children.

**Supplementary Information:**

The online version contains supplementary material available at 10.1186/s13104-021-05500-3.

## Introduction

Thinness in children received little attention, when compared to obesity which is rising in developed and developing countries [1, 2]. Studies have shown that there is a double burden of childhood obesity and thinness, especially in modern societies [3–5]. Therefore, the impact of thinness and its sequelae need to be studied, especially the effects of delayed puberty.

Reports in the Middle East on children with impaired growth, consist of descriptive surveys with little attention to the impact of poor nutrition on children [6–9]. In one study, one-third of thin children remained thin as adults [10]. Thus underweight may be associated with lifelong sequelae [11–13]. In addition, low body fat delays puberty while excess body fat promotes early puberty [14].

Efforts to tackle obesity might adversely increase thinness. And it is unknown if childhood thinness has been affected by increasing obesity. Thinness can result from poor feeding, eating practices, conditions such as celiac disease, cystic fibrosis, or malabsorptive disorders [15]. Furthermore, it may result in long-term hazards for child development, stunting, weakening of the immune system, osteoporosis, anemia, and fertility problems later in life [16–19].

In females, a percentage of fat ≥ 17% of the total body weight is needed for menarche and ≥ 22% for the maintenance of regular cycles [20, 21]. This link between body fat and puberty is mediated by factors, including leptin and the aromatization of androgens to estrogens in adipose tissue [22]. The so-called ‘food intake signal’ describes a caloric intake per 100 g of body weight that becomes a *de fact*o signal for puberty [23].

The effects of undernutrition on growth and puberty are largely reversible. In one study on children with self-imposed restrictions of caloric intake for fear of obesity, linear growth and sexual development resumed after adequate caloric intake [24]. These results should encourage health caregivers to treat underweight children early. Ascertaining the local prevalence of childhood thinness is necessary, especially when it may inadvertently be worsened by measures introduced to tackle childhood obesity. This is supported by a study evaluating children for short stature and/or delayed puberty which found, that growth failure was attributed to malnutrition secondary to self-imposed restriction of caloric intake arising from a fear of obesity [24].

Defining thinness is challenging, as BMI classification differs among the three commonly used scales, International Obesity Task Force (IOTF), the World Health Organization (WHO) and the Centers for Disease Control (CDC), with none claiming clear superiority [25, 26].

The objectives of this study are investigate the prevalence of BMI groups in a cohort of schoolchildren using the three standards, and to calculate, using the IOTF standard, the prevalence of thinness by age and sex, including its trend by age, and its relationship with height velocity.

## Main text

### Methods

This cross-sectional population-based observational study was conducted during 2014–2015 in Ras Al-Khaimah (RAK), United Arab Emirates (UAE), in 29,410 schoolchildren. A subset of previously reported cohort [2].

The measurements were carried out by trained school nurses (certified and registered under the Ministry of Health). In each school there was one nurse that took the measurements, as an integral component of the governmental school annual health assessment as previously described [2, 27]. Anthropometric measurements were done as previously described in detail [2, 27]. Measurements accuracy was regularly checked by head nurses and school physicians. Ethical approval (REC reference number: 12/2016-F) was granted by RAK-Research Ethics Committee and the consent was waived as the data collected was part of a regular school health assessment program and the collected data was anonymized.

To calculate age-group annual height velocity, children were divided into consecutive one-year age groups, and for each group, the height velocity was the average height at that age minus the average height immediately preceding group. For example, the nine-year group comprised all children between eight and nine years of age. The annual height velocity for that age group was the difference between the mean height for that group minus the mean height of the eight-year group. The mean group annual height velocity and the standard error of the mean (SE) were reported as cm/year.

The BMI was calculated as weight (kg)/height (m)^2^. The BMI classification was as defined by the IOTF, WHO and CDC standards (Additional file 1) and as described by our group [2].

We reported the BMI and the prevalence of thinness by the three standards. Using the IOFT classification, through a univariate and multivariate logistic regression analysis of the prevalence of thinness by age and sex, as well as the trend of thinness by age. We also reported the association of thinness with height velocity, by age and by sex.

The continuous variables were presented as mean ± SD or SE and the variables were compared using *t*-tests between the two groups, or with an analysis of variance (ANOVA) with groups of three or more. Categorical variables were expressed as numbers and percentages with 95% confidence intervals (CI) and were compared using a chi-squared test, including their unadjusted odds ratios (OR) and 95% CIs. Their adjusted ORs were calculated with a logistic regression model that included possible confounders. A test for trend in the prevalence of thinness with age was also performed using the Cochran-Armitage test for trend. Statistical analysis was carried out using Stata software (version 14; StataCorp LLC, College Station, TX USA) and statistical significance was defined by a two-tailed *P* < 0.05.

### Results

A total of 29,410 children were enrolled, half of whom were females (n = 14,849). Their mean (SD) age was 10.4 (3.8) years, (median 10.2 years, range: 3–19 years). Ninety-two percent (n = 27,000) were UAE citizens and lived in urban areas. The majority of schoolchildren (82%) were between grade one and nine,and (60%) between grade one and five.

1. BMI classification
  The prevalence of BMI classification by the three standards and by sex and age are shown in Additional files 2, 3, 4. Using IOTF standards (Table 1), the proportion of thinness increased in children seven to nine years compared to that in children four to six years after which it decreased with age, reaching 8–12% in female and male adolescents (*P* < 0.001; chi-squared test). Overweight and obesity increased with age, while the proportion of children with normal BMI did not markedly change (Additional file 2). The prevalence of BMI classification by IOTF are shown in Tables 1, 2 and Additional file 2. Similar patterns were found with the WHO and the CDC standards (Additional files 1, 2, 3, 4, 5, 6) with each reaching the level of statistical significance (*P* < 0.001; chi-square test).
2. Thinness
  The prevalence of thinness by the three standards showed a significant decrease between the highest prevalence using the IOTF standard (21.6%), and the lowest (11.0%) using the WHO standard (Additional file 5; *P* < 0.001). The calculated prevalence of thinness by age, was compared between the three standards (Fig. 1). In both sexes and at all ages, that prevalence was the highest by the IOTF standard and the lowest by the WHO standard (*P* = 0.03; ANOVA). The difference in the prevalence between the three standards was the lowest between the ages of 7–17 years.
  The unadjusted ORs of thinness by age, using the IOTF standard, was 0.67 (95% CI 0.66–0.69; P < 0.001) and by sex it was 0.91 (95% CI 0.86–096). When adjusted for in a logistic regression model, only age remained significantly associated with thinness (adjusted OR: 0.86; 95% CI 0.85–0.87; *P* < 0.001). Thinness trend by age showed a significant decline in prevalence with advancing age (Additional file 6; *P* < 0.001).

**Fig. 1:**
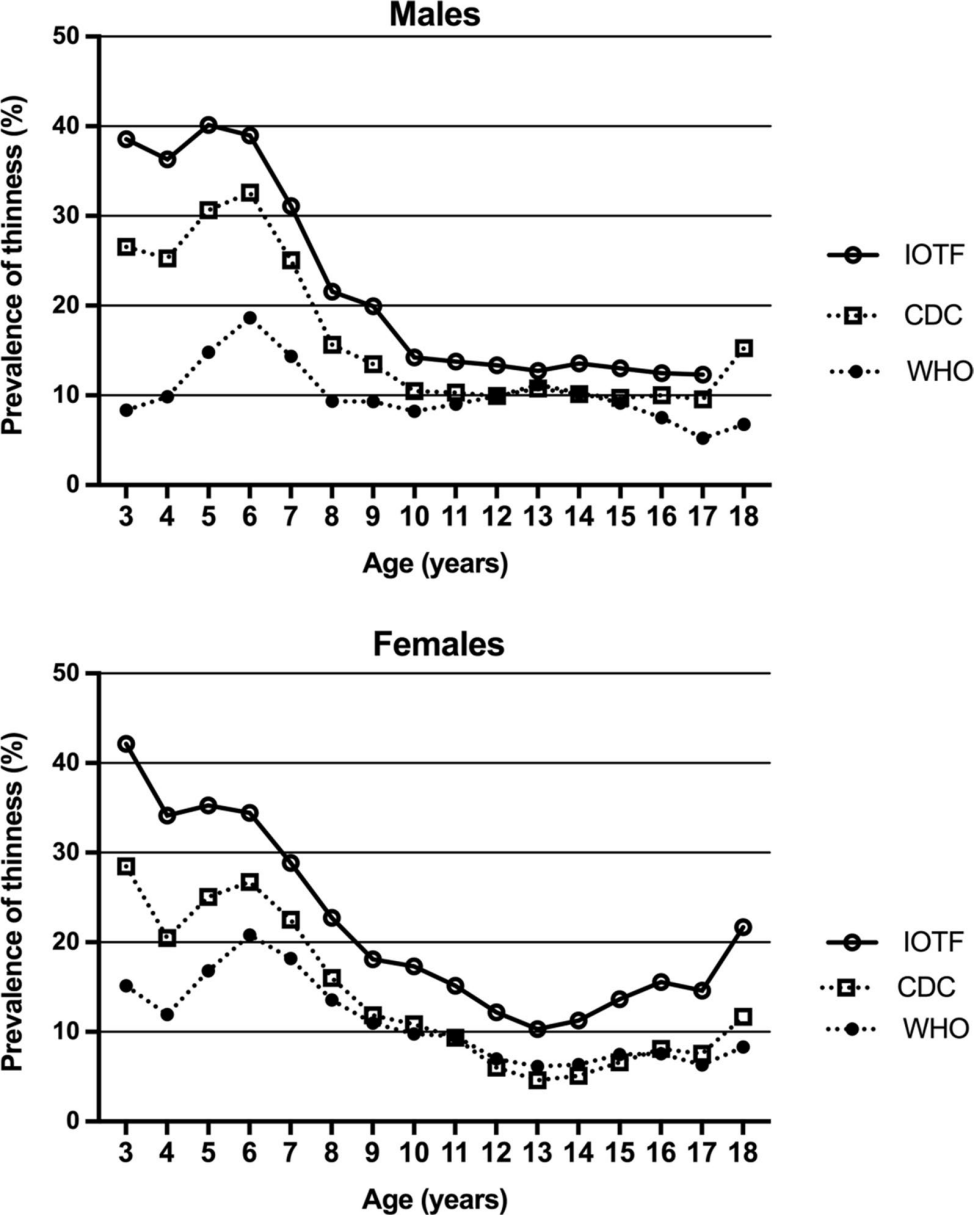
Prevalence of thinness (percent) by age in both sexes as derived from the three growth standards (International Obesity Task Force, World Health Organization, and Centers for Disease Control)
3. Annual peak height velocity
  The annual group height velocity for each IOTF-BMI group (Table 1) was analyzed (Additional file 2). The peak group height velocity consistently showed significant differences of at least 10% (*P* < 0.05). In children under the age of 13 years, the peak group height velocity was significantly lower in thin children than in those with excess body fat (*P* = 0.001) and occurred at an older age in thin children (15 years in males, *P* = 0.4; 13 years in females, *P* = 0.02) when compared to normal children and those with excess body fat (Table 1).

**Table 1:** Body mass index distribution per the IOTF classification as a function of age and sex

	Thin (%)	Overweight (%)	Obese (%)	Extremely Obese (%)	Normal (%)
Females					
4–6 years (*n* = 1,976)	23.2	5.9	3.2	8.7	14.5
7–9 years (*n* = 3,352)	32.4	15.4	15.5	18.2	23.6
10–12 years (*n* = 3,492)	19.9	29.9	29.5	21.7	23.2
13–15 years (*n* = 3,179)	12.1	29.5	30.5	27.8	21.5
16–18 years (*n* = 2,513)	12.4	19.4	21.3	23.7	17.2
*Peak (age) height velocity*	*6.5* ± *0.3 cm/years (at 13 years)*	*7.0* ± *0.3 cm/years at 11 years)*	*8.0* ± *0.3 cm/years (at 10 years)*	*7.1* ± *0.3 cm/years (at 11 years)*	*6.5* ± *0.3 cm/years (at 11 years)*
Males					
4–6 years (*n* = 2,064)	25.2	4.7	2.8	7.8	15.5
7–9 years (*n* = 3,683)	35.9	16.7	14.9	17.3	27.0
10–12 years (*n* = 3,649)	18.7	31.5	30.9	21.7	26.2
13–15 years (*n* = 2,902)	12.2	29.5	31.2	28.4	18.3
16–18 years (*n* = 1,988)	8.0	17.5	20.4	24.8	12.9
*Peak (age) height velocity*	*7.7* ± *0.3 cm/years (at 15 years)*	*6.3* ± *0.3 cm/years (at 12 years)*	*6.7* ± *0.3 cm/years (at 12 years)*	*6.4* ± *0.3 cm/years (at 11 years)*	*6.7* ± *0.3 cm/years (at 12–14 years)*

Peak group height velocity (mean ± standard error) and age at the peak height velocity after the age of 10. IOTF: International Obesity Task Force

**Table 2:** Prevalence of BMI groups in 29,410 children by the IOTF classification

	Total participants n = 29,410			Males n = 14,561			Females n = 14,849			*P value
	Number	Prevalence (%)	95% CI	Number	Prevalence (%)	95% CI	Number	Prevalence (%)	95% CI	
Extremely obese	1,454	4.9	4.6, 5.2	719	4.9	4.6, 5.3	735	4.9	4.6, 5.3	0.003
Obese	2,492	8.4	8.1, 8.8	1,277	8.8	8.3, 9.2	1,215	8.2	7.7, 8.6	
Overweight	4,622	15.7	15.3, 16.1	2,223	15.3	14.7, 15.8	2,399	16.1	15.5, 16.7	
Normal	14,490	49.3	48.6, 49.8	7,090	48.7	47.8, 49.5	7,400	49.8	49.0, 50.6	
Underweight	6,352	21.6	21.1, 22.1	3,252	22.3	21.7, 23.0	3,100	20.9	20.2, 21.5	

BMI: body mass index; IOTF: International Obesity task Force; CI: confidence intervals

*chi-squared test

The peak height velocity was the highest (8.0 ± 0.3 cm/year) in obese females at the age of 10 years and in obese males (6.7 ± 0.3 cm/year) at the age of 12 years. It was the lowest (6.5 ± 0.3 cm/year) at 13 years in thin females and at 11 years in females with normal BMI, while in obese males it occurred at the age of 12 years (6.3 ± 0.3 cm/year). In thin children, the height velocity was not significantly associated with age in males (*P* = 0.09) or in females (*P* = 0.17). The annual group peak height velocities for each BMI group by WHO and CDC classification are shown in Additional files 3, 4.

### Discussion

This study showed that, in affluent UAE society with increasing childhood obesity, the prevalence of thinness was higher in younger children, mainly between the ages of three and eight years, reaching 20–40%. With increasing age, thinness decreased in prevalence while it regularly increased for obesity. These findings were consistent in both sex and across the three growth standards. The prevalence of thinness in other countries, such as Jordan and Romania was reported to be much lower (5.7–8.5%) [6]. However, these studies did not include children under six years, where thinness is relatively common [6]. While our results are closer to reports from Oman (17.9%) and Indonesia (21–34%) [28–30], further studies are needed to evaluate if these results are generalizable to other countries.

Our data shows that thinness decreases with age. In a previous study, the frequency of underweight remained constant with advanced childhood age [30]. Differences in study design, population characteristics and environmental factors might explain such variations. It is, however, encouraging to see that thinness decreases with age in our population, confirming previously reported reversibility [24].

Group peak height velocity (calculated after the age of 10 years) was delayed by one to three years in thin children, regardless of their sex, and was higher in children with excess body fat. These results confirm previous studies in which underweight prepubertal children had a delayed peak height velocity and entered puberty significantly later than those with an elevated BMI [31]. Other reports also support our findings by demonstrating that, during prepubertal years, lean children have a lower height velocity compared to those with obesity [32].

## Limitation

The lack of data on children’s nutrition, breastfeeding duration, socioeconomic level and parents’ educational is one of this study limitations. In addition, being a descriptive cross-sectional study, there were no data on the long-term follow-up of outcomes of interest, including puberty which need to be included in future studies [33, 34].

Although the pubertal stage was not evaluated in our study, measuring the peak height velocity, as a surrogate marker, confirms the negative association of thinness on pubertal development. Overall, thin children had a peak group height velocity later than normal children did, while overweight and obese children had their growth spurt earlier, confirming earlier reports [14, 20–22]. We acknowledge that, because of cultural sensitivities, assessing the children’s Tanner pubertal stage could not be performed. In addition, we believe that it would have been inappropriate to use the Tanner standards, as, unlike our cross-sectional study, they were developed from longitudinal studies. Instead, we chose to calculate group height velocity as a surrogate marker for the onset of puberty.

Because we studied the anthropometric measurements of each child once, the association of thinness with decreased height velocity, while true at a group level, was not necessarily true in the individual child, neither does it necessarily demonstrate causality. In addition, it unknown whether the observed decrease in the prevalence of thinness, amid a simultaneous increase in the rate of obesity reflects a progressive change in the BMI trajectory in individual children or, instead, represents two entirely separate subgroups of youngsters. Longitudinal studies are needed to understand the dynamics of these growth profiles.

Despite these limitations, this study confirms the need for health professional to consider, in addition to obesity, thin children as a public health concern. Studies should also evaluate if, in addition, it may potentially and inadvertently be worsened by increased awareness of obesity and the introduction of public health measures to tackle it.

## Supplementary Information

**Additional file 1**. BMI classification by the CDC, WHO and IOTF standards. BMI classification by the CDC, WHO and IOTF standards.

**Additional file 2. **Body mass index classification by the International Obesity Task Force standards and mean annual group height velocity. Distribution of body mass index classification (panel A and B) and mean annual group height velocity (panel C and D) as a function of age and sex. The standard errors of all mean height velocity measurements ranged from 0.3 to 0.5 cm/year and are not displayed in the graphs.

**Additional file 3. **Body mass index by the World Health Organization standards and mean annual group height velocity..Distribution percentage of body mass index classification (panel A and B) and mean annual group height velocity (panel C and D) as a function of age and sex. The standard errors of all mean height velocity measurements ranged from 0.3 to 0.5 cm/year and are not displayed in the graphs.

**Additional file 4. **Body mass index by the Centers for Disease Control standards and mean annual group height velocity. Distribution percentage of body mass index classification (panel A and B) and mean annual group height velocity (panel C and D) as a function of age and sex. *The standard errors of all mean height velocity measurements ranged from 0.3 to 0.5 cm/year and are not displayed in the graphs*.

**Additional file 5. **Comparison of the prevalence of thinness based on the IOTF, CDC, and WHO classifications (n = 29,410). IOTF: International Obesity Task Force; CDC: Centers for Disease Control and Prevention; WHO: World Health Organization; *chi-squared for R x C table.

**Additional file 6. **Prevalence of thinness by age using the IOTF BMI classification. BMI; body mass index; IOTF: International Obesity Task Force; CI confidence intervals; *chi-squared test.

## Data Availability

The datasets used and/or analysed during the current study available from the corresponding author on reasonable request.

## Abbreviations

ANOVA: Analysis of variance
BMI: Body mass index
CDC: Centers for disease control
CI: Confidence interval
IOTF: International obesity task force
OR: Odds ratio
RAK: Ras Al Khaimah
SD: Standard deviation
SE: Standard error
UAE: United Arab Emirates
WHO: World Health Organization
