# Supplementary material for: Prevalence of thinness and its effect on height velocity in schoolchildren
Source: BMC Res Notes. 2021 Mar 16;14:98. doi: 10.1186/s13104-021-05500-3 (PMC7962207; doi:10.1186/s13104-021-05500-3)
Supplement: Supplementary file 1 — Additional file 1. BMI classification by the CDC, WHO and IOTF standards. BMI classification by the CDC, WHO and IOTF standards. [file 13104_2021_5500_MOESM1_ESM.docx]

**Additional File 1**

**BMI classification by the CDC, WHO and IOTF standards**

|  | **BMI (kg/m2) or percentile** | **Classification** |
| --- | --- | --- |
| **CDC** |  |  |
|  | ≥ 95^th^ percentile | Obese |
|  | 85^th^ to < 95^th^ percentile | Overweight |
|  | 5^th^ to < 85^th^ percentile | Healthy Weight |
|  | < 5th percentile | Underweight |
| **WHO** |  |  |
|  | ≥ 30 kg/m^2^ | Obesity |
|  | ≥ 25 to 29.9 kg/m^2^ | Overweight |
|  | ≥ 18.5 to 24.9 kg/m^2^ | Normal weight |
|  | < 18.5 kg/m^2^ | Underweight |
|  | < 16.5 kg/m^2^ | Severely underweight |
| **IOTF** |  |  |
|  | ≥40 kg/m^2^ | Severely Obese |
|  | ≥30 kg/m^2^ | Obese |
|  | ≥25 kg/m^2^ | Overweight (including obesity) |
|  | 18.5-24.99 kg/m^2^ | Healthy Weight |

CDC: Center for Disease Control and prevention; WHO: World health Organisation; IOTF: International Obesity task force
