# Supplementary figures and images for: Prevalence of thinness and its effect on height velocity in schoolchildren
Source: BMC Res Notes. 2021 Mar 16;14:98. doi: 10.1186/s13104-021-05500-3 (PMC7962207; doi:10.1186/s13104-021-05500-3)

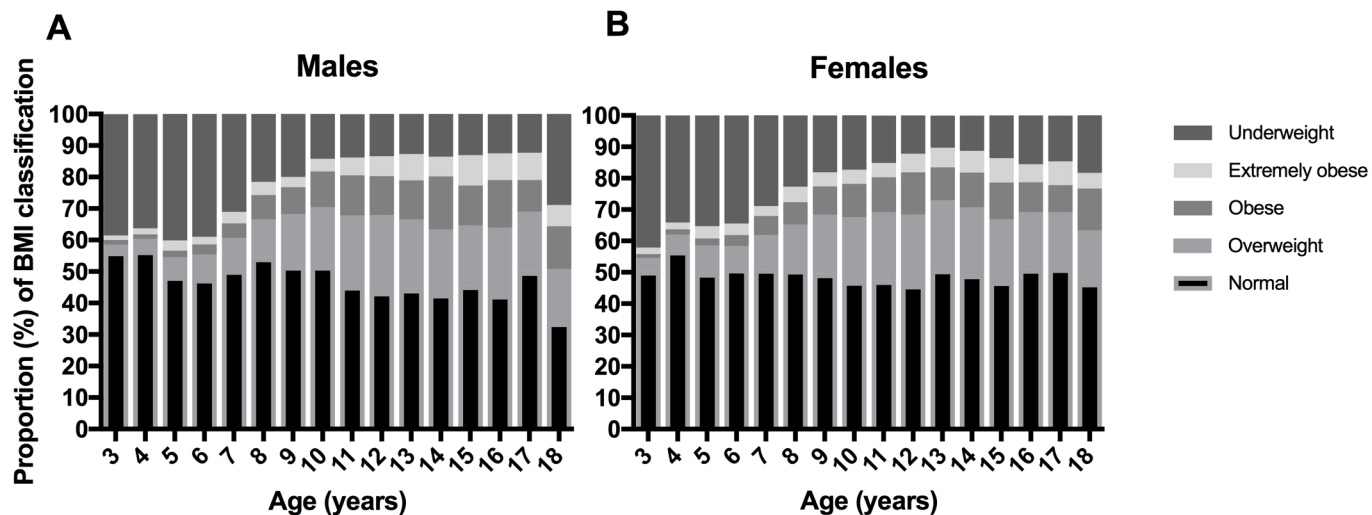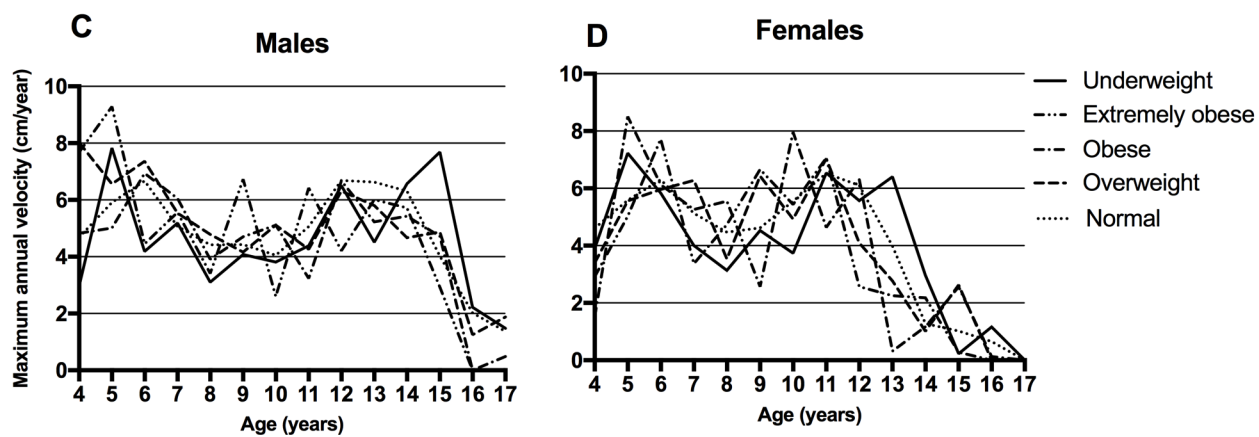

Supplement: Supplementary file 2 — Additional file 2. Body mass index classification by the International Obesity Task Force standards and mean annual group height velocity. Distribution of body mass index classification (panel A and B) and mean annual group height velocity (panel C and D) as a function of age and sex. The standard errors of all mean height velocity measurements ranged from 0.3 to 0.5 cm/year and are not displayed in the graphs. [file 13104_2021_5500_MOESM2_ESM.pdf]

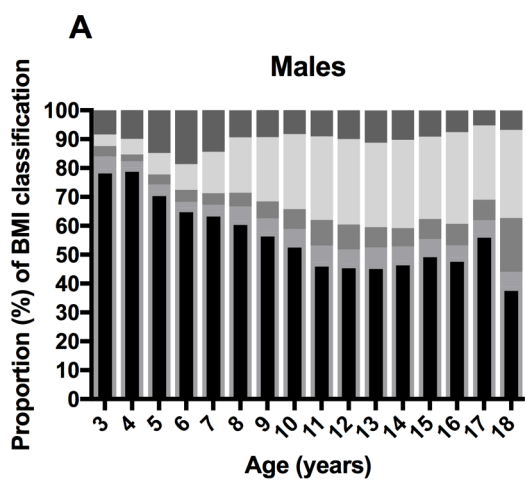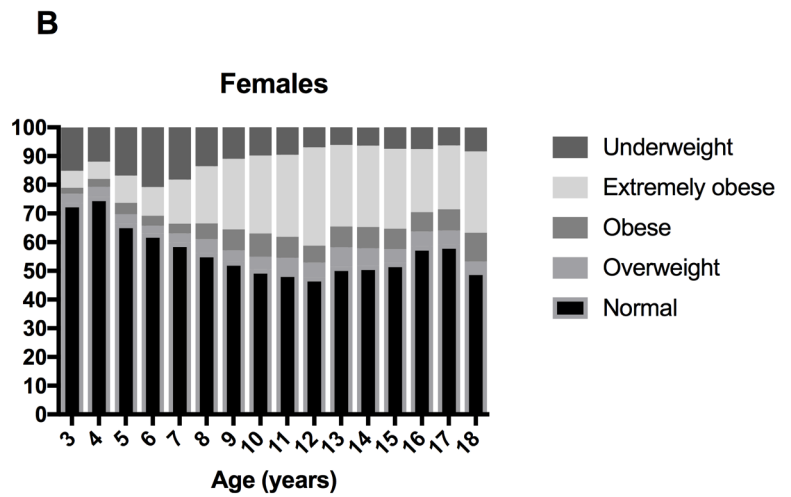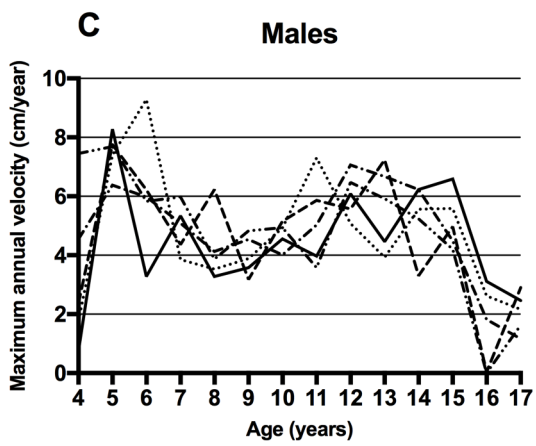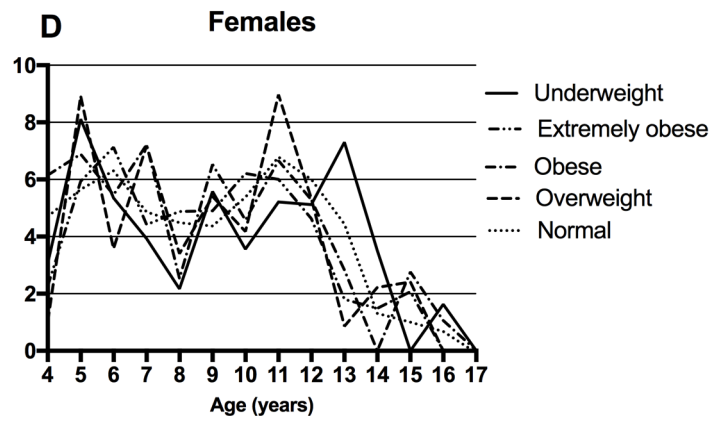

Supplement: Supplementary file 3 — Additional file 3. Body mass index by the World Health Organization standards and mean annual group height velocity..Distribution percentage of body mass index classification (panel A and B) and mean annual group height velocity (panel C and D) as a function of age and sex. The standard errors of all mean height velocity measurements ranged from 0.3 to 0.5 cm/year and are not displayed in the graphs. [file 13104_2021_5500_MOESM3_ESM.pdf]

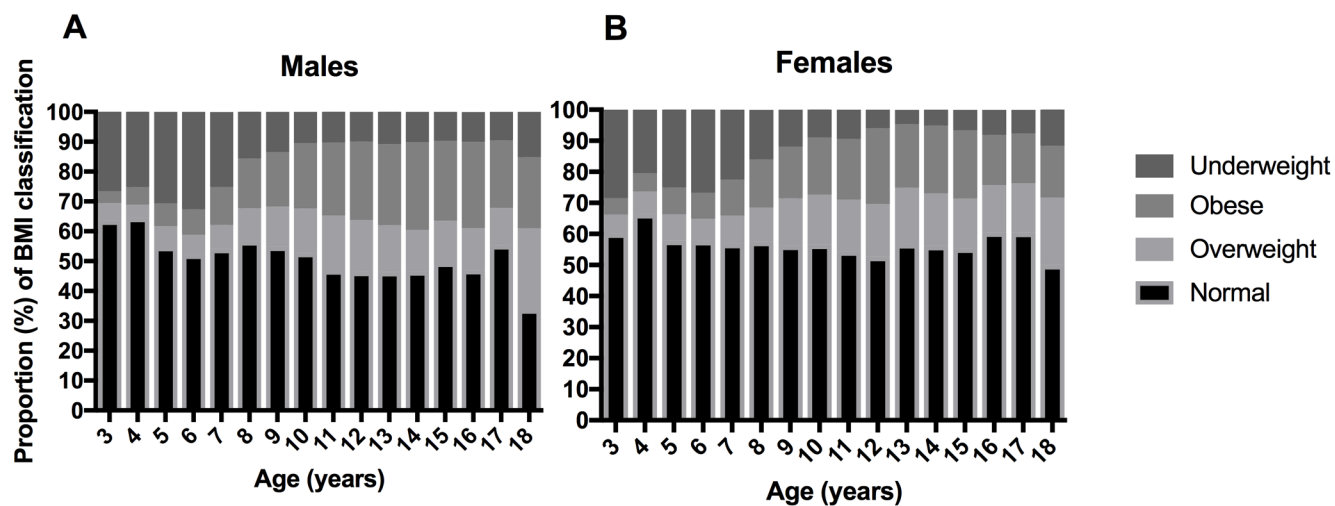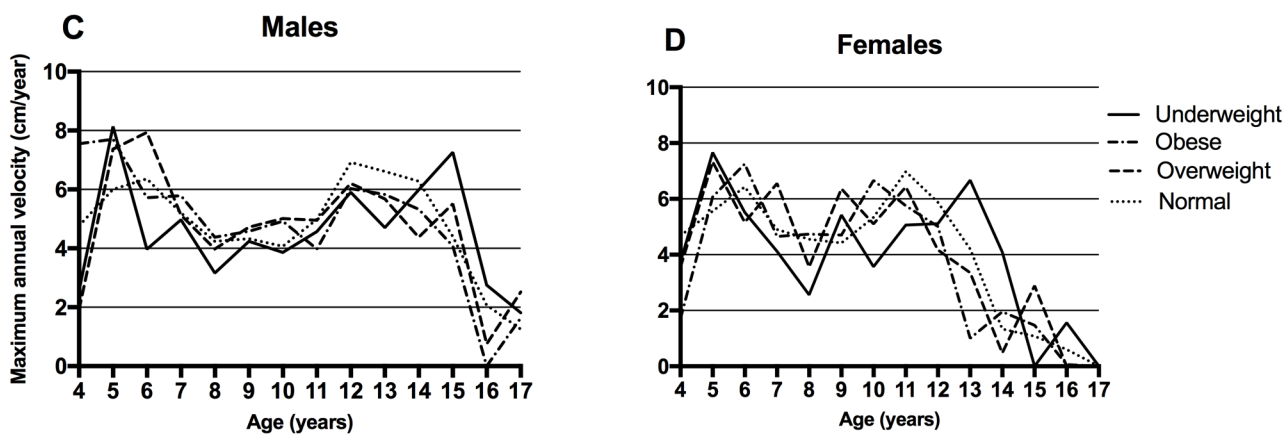

Supplement: Supplementary file 4 — Additional file 4. Body mass index by the Centers for Disease Control standards and mean annual group height velocity. Distribution percentage of body mass index classification (panel A and B) and mean annual group height velocity (panel C and D) as a function of age and sex. The standard errors of all mean height velocity measurements ranged from 0.3 to 0.5 cm/year and are not displayed in the graphs. [file 13104_2021_5500_MOESM4_ESM.pdf]
