# Supplementary material for: Prevalence of thinness and its effect on height velocity in schoolchildren
Source: BMC Res Notes. 2021 Mar 16;14:98. doi: 10.1186/s13104-021-05500-3 (PMC7962207; doi:10.1186/s13104-021-05500-3)
Supplement: Supplementary file 5 — Additional file 5. Comparison of the prevalence of thinness based on the IOTF, CDC, and WHO classifications (n = 29,410). IOTF: International Obesity Task Force; CDC: Centers for Disease Control and Prevention; WHO: World Health Organization; *chi-squared for R x C table. [file 13104_2021_5500_MOESM5_ESM.docx]

| **Additional File 5.** Comparison of the prevalence of thinness based on the IOTF, CDC, and WHO classifications (n=29,410) | | | | |
| --- | --- | --- | --- | --- |
|  | **Thin children (n)** | **Prevalence (%)** | **95% CI** | ****P* value** |
| IOTF | 6,352 | 21.6 | 21.1, 22,1 | <0.001 |
| CDC | 4,483 | 15.2 | 14.8, 15.6 |  |
| WHO | 3,252 | 11.0 | 10.7, 11.4 |  |
| IOTF: International Obesity Task Force; CDC: Centers for Disease Control and Prevention; WHO: World Health Organization; *chi-squared for R x C table | | | | |
