# Supplementary material for: Prevalence of thinness and its effect on height velocity in schoolchildren
Source: BMC Res Notes. 2021 Mar 16;14:98. doi: 10.1186/s13104-021-05500-3 (PMC7962207; doi:10.1186/s13104-021-05500-3)
Supplement: Supplementary file 6 — Additional file 6. Prevalence of thinness by age using the IOTF BMI classification. BMI; body mass index; IOTF: International Obesity Task Force; CI confidence intervals; *chi-squared test. [file 13104_2021_5500_MOESM6_ESM.docx]

| Additional File 6**.** Prevalence of thinness by age using the IOTF BMI classification | | | | | |
| --- | --- | --- | --- | --- | --- |
| **Age (completed) years** | **Children** | **Thin children** | **Prevalence (%)** | **95% CI** | ****P* value** |
| 3 | 612 | 248 | 40.5 | 36.6–44.5 | <0.001 |
| 4 | 1,773 | 625 | 35.2 | 33.0–37.5 |  |
| 5 | 2,267 | 856 | 37.7 | 35.7–39.8 |  |
| 6 | 2.370 | 873 | 36.8 | 34.9–38.8 |  |
| 7 | 2,320 | 697 | 30.0 | 28.1–31.9 |  |
| 8 | 2,345 | 519 | 22.1 | 20.4–23.8 |  |
| 9 | 2,449 | 467 | 19.0 | 17.5–20.7 |  |
| 10 | 2,460 | 387 | 15.7 | 14.3–17.2 |  |
| 11 | 2,232 | 323 | 14.5 | 13.0–15.9 |  |
| 12 | 2,113 | 270 | 12.8 | 11.4–14.3 |  |
| 13 | 2,099 | 240 | 11.4 | 10.1–12.9 |  |
| 14 | 1,869 | 230 | 12.3 | 10.8–13.9 |  |
| 15 | 1,644 | 220 | 13.4 | 11.7–15.1 |  |
| 16 | 1,745 | 248 | 2.1 | 1.8–2.4 |  |
| 17 | 993 | 135 | 13.6 | 11.5–15.9 |  |
| 18 | 119 | 14 | 11.7 | 6.5–18.9 |  |
| Total | 29,410 | 6,352 | 21.6 | 21.1, 22.1 |  |
| BMI; body mass index; IOTF: International Obesity Task Force; CI: confidence intervals; *****chi-squared test | | | | | |
